# High dose interstitial brachytherapy for treatment of carcinoma of the lip as an alternative to surgery: a case report

**DOI:** 10.3332/ecancer.2021.1297

**Published:** 2021-09-30

**Authors:** Percy Torres-Quispe, Lissett Jeanette Fernández-Rodríguez, Yaowen Zhang, Angeles Rovirosa-Casino

**Affiliations:** 1 Department of Oncology and Radiotherapy, Hospital Nacional Edgardo Rebagliati Martins, Av. Edgardo Rebagliati 490, Jesús María, Lima 15072, Perú; 2 Universidad Nacional San Antonio Abad del Cusco, Av. de La Cultura 773, Cusco 08000, Perú; 3 Diagnosis and Treatment Assistance Department, Hospital de Alta Complejidad Virgen de la Puerta, M.Bastidas 309, La Esperanza, La Libertad 13013, Perú; 4 Universidad Privada Antenor Orrego, Avenida América Sur 3145, Trujillo, La Libertad 13008, Perú; 5 Radiation Oncology Service, Hospital Clinic i Provincial de Barcelona, C. de Villarroel, 170, Barcelona 08036, Spain; 6 Fonaments Clinics Department, Faculty of Medicine, Universitat de Barcelona, Casanova, 143, Barcelona 08036, Spain; * Current affiliation: Department of Medicine, Hospital de Alta Complejidad Virgen de la Puerta, M.Bastidas 309, La Esperanza, La Libertad 13013, Perú; † Current affiliation: Cancer Center, Henan Provincial People's Hospital, Zhengzhou 450008, China; a https://orcid.org/0000-0003-1434-1925; b https://orcid.org/0000-0002-4357-4261; c https://orcid.org/0000-0002-2379-6174; d https://orcid.org/0000-0002-7832-334X

**Keywords:** brachytherapy, lip neoplasm, iridium radioisotopes, squamous cell carcinoma

## Abstract

Lip cancer (LC) is the most common cancer of the oral cavity and is the second most common in the head and neck. Brachytherapy (BT) is a good treatment option for this type of cancer because it administers high doses of radiation to the target. BT has similar cure and survival rates as surgery for the same type of cancer, but it often produces more favourable aesthetic results, especially when the tumour is treated in its early stages. We present a case of a patient diagnosed with stage II lower LC who refused surgical treatment. Instead, high-dose-rate interstitial BT was performed with ^192^Ir. A 2-year post-treatment follow-up indicated there were neither serious complications nor recurrence of cancer during that period.

## Background

Lip cancer (LC) is the most common malignancy of the oral cavity and is the second most common in the head and neck. It has a worldwide age-standardised incidence of 5.8 per 100,000 persons per year and it affects predominately males [1]. LC is associated with high solar exposure, tobacco and alcohol use, poor oral hygiene, lip trauma, immunosuppression and precancerous lesions [2–4]. Nearly all (90%) LC is localised in the lower lip (LL), and the type of cancer most found is well-differentiated squamous cell carcinoma, followed by basal cell tumours [4].

LC tends to form tumours that are easily visualised and localised, which grow slowly, permitting early diagnosis. Therefore, LC has a high rate of survival among those treated [3]. Lymphatic spreading in the cancer’s early stages rarely occurs. At diagnosis, only 5%–10% of cases have lymph node metastasis, with submental (level Ia) and submandibular (level Ib) lymph nodes being the most affected [4]. More distant metastases are also rare but have been found with more advanced tumours. Prognostic factors include tumour size, lymph node infiltration, perineural and lymphovascular invasion and age, with the youngest having poorer prognoses [4–6]. The 5-year survival rate is between 90% and 95% at stage T1, 75%–85% at stage T2, with a large decrease for T3–T4 [3].

Both surgery and brachytherapy (BT) play important roles in LC treatment, given that these treatments alone or in combination with external radiotherapy are indicated in over 90% of cases [4]. High-dose-rate interstitial brachytherapy (HDRIB) is frequently used to treat LC. This procedure involves placing applicators in the tumour and then repeatedly placing a radiation source within the applicator. The applicators are removed upon treatment completion [5]. ^192^Ir is frequently used in HDRIB due to its high specific activity and photon energy, ensuring homogenous treatment of the target.

HDRIB has a number of advantages over a more radical surgical approach. It is painless when used with local anaesthesia or intravenous analgesics, provides a focused dose to the tumour bed with minimal damage to healthy tissue and the rest of the body, has low toxicity (no permanent contact with radioisotope source) and preserves anatomy. Furthermore, HDRIB has a good aesthetic outcome, reduces the risk of surgical microstomia and reduces radiation exposure to health personnel [5, 7–9]. However, developing a treatment plan, acquiring radioisotopes and appropriate software, and ingrained traditional approaches to treating LC present a challenge to adopting HDRIB in hospitals located in less developed countries, like Peru. Therefore, we present one of the first cases of LC treated with HDRIB in Peru, which required a collaborative approach between the patient and physicians to increase treatment options for LC.

### Patient information

**Figure 1.**
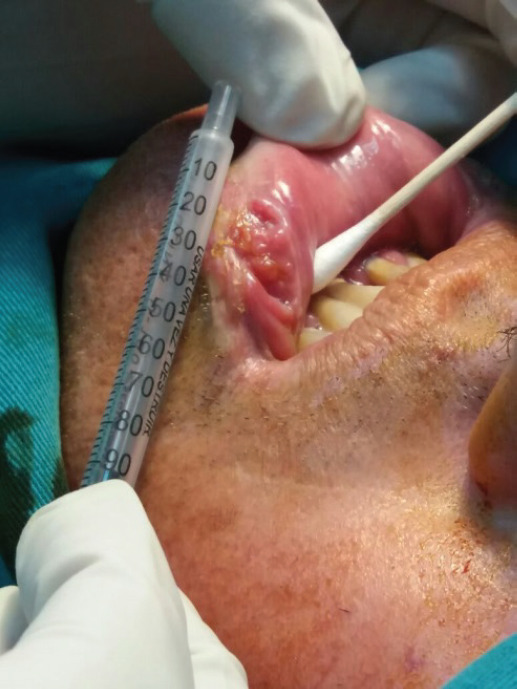
Photograph of the ulcerative mucosal lesion of the lower left lip before treatment.

A 53-year-old male from Lima, Peru, with a six cigarette-per-day smoking habit for 20 years was admitted for evaluation of an ulcerative lesion on the left side of the LL. The lesion was previously diagnosed as well-differentiated infiltrating squamous cell carcinoma in a different health centre. He stated that the lesion had been present for 2 years. A 1.5 × 2.5 cm nodule adjacent to the lesion was revealed upon palpitation (Figure 1). No enlarged lymph nodes or adenopathies were found upon palpation or ultrasound. The patient had no additional pathological antecedents of interest.

A computed tomography (CT) scan revealed premandibular cutaneous laminar thickening of a likely reactive inflammatory nature and normal lungs. Swollen lymph nodes were also observed. Microscopic review of biopsies confirmed the diagnosis. The cancer was staged at T2N0M0 according to TNM guidelines [4].

The patient was given a choice of surgery or HDRIB to treat the neoplasm. He opted for HDRIB because he preferred to avoid cuts or scars on his face. The patient was admitted to the hospital and given clindamycin (600 mg) and bicarbonate mouth rinses every 8 hours.

**Figure 2.**
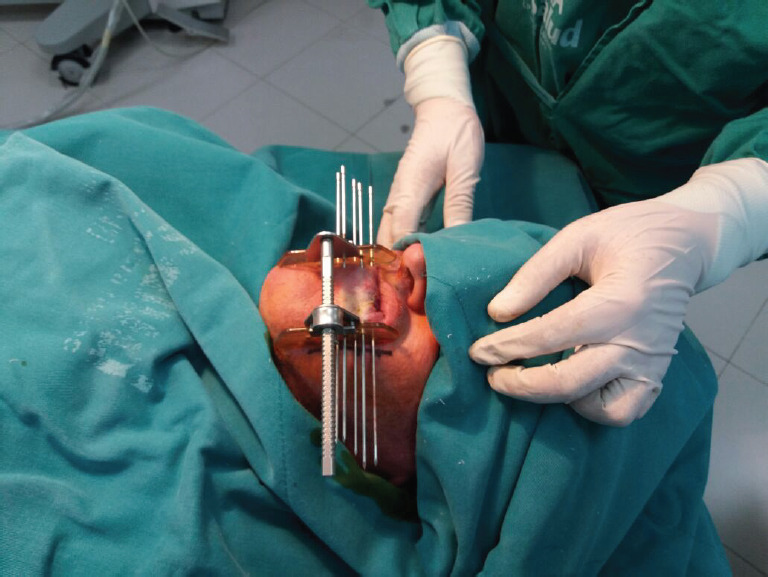
Placement of metal needles according to the Paris system is assisted by clear plastic templates held in place by a metal frame.

**Figure 3.**
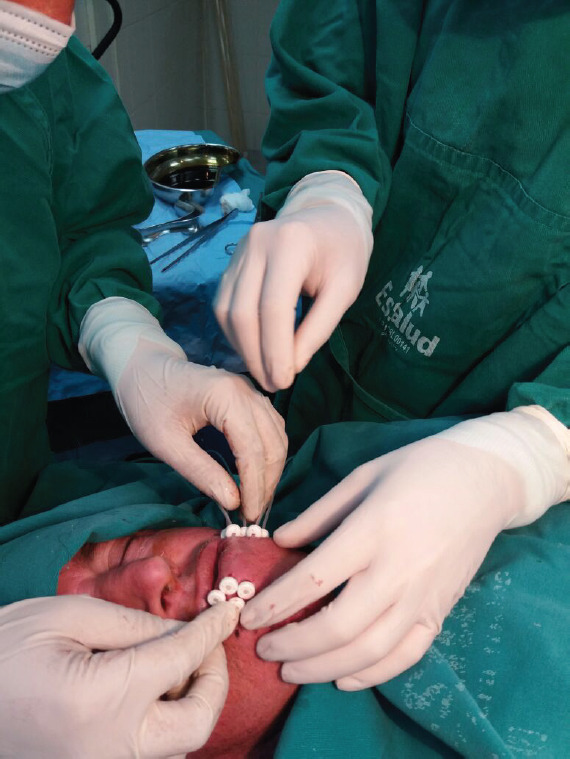
Stabilization of BT catheters using radiolucent buttons in the LL.

Under local anaesthesia, five equidistant metallic needles were inserted through the left side of the LL with the assistance of plastic templates (Figure 2). Plastic catheters were pulled through the tissue as the needles were withdrawn. The catheters were stabilized in the patient with the use of white radiolucent buttons (Nucletron, 1.9 mm) (white plastic circles, Figure 3). Placement of the needles and catheters followed the Paris system [4, 8].

**Figure 4.**
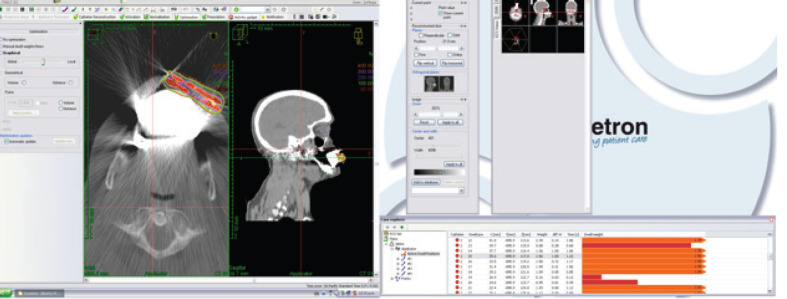
Simulation of HDRIB with ^192^Ir for irradiation of the patient’s tumour.

After the catheters were inserted, a CT image was taken of the lesion, adjacent nodule and surrounding tissues. This image was used to plan HDRIB treatment (Figure 4, Table 1). For simulation, the gross tumoural volume included both the lesion and nodule, and the clinical tumoural volume (CTV) was considered to be the same with a 1 cm margin. Radiation dose was administered according to a dosimetric study that considered the surrounding organs [10].

Following the treatment plan, ^192^Ir pellets were placed in the catheters for a series of nine fractions over 5 days. On the first day of treatment, the patient received one fraction of 5.00 Gy. For the next 4 days, two daily fractions of 5.00 Gy were administered at an interval of 6 hours providing a total equieffective dose (EQD2)_(α/β = 10)_ of 56 Gy. A dental shield made of lead covered in paraffin was used to protect the gingiva during the HDRIB treatments. After HDRIB was complete, the plastic catheters were removed.

One month post treatment, the patient received his first follow-up. The patient had localised acute radiation toxicity with bleeding ulcerative mucositis at the treatment site. He was prescribed analgesics, anti-inflammatory drugs and a mouthwash to treat his symptoms. The mouthwash was prepared in-house and contained 30 mL lidocaine gel (2%), 12 mL nystatin (100,000 IU/mL), 60 mL sulfamethoxazole and trimethoprim (40 mg/mL and 8 mg/mL), 120 mL of aluminium hydroxide/magnesium hydroxide with simethicone and 1 L of distilled water.

**Figure 5.**
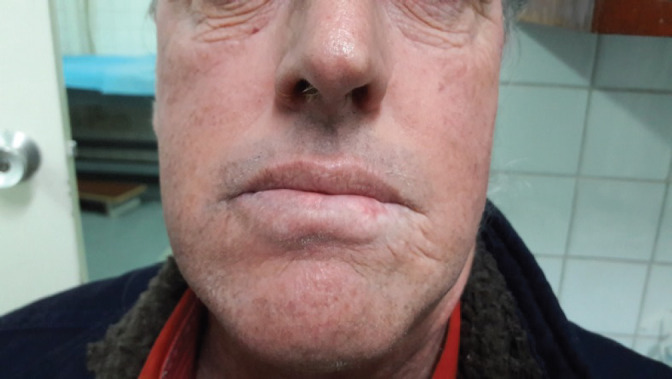
Two-year post-treatment result, indicating mild atrophy and hypopigmentation. The patient was pleased with the aesthetic result.

At his second follow-up, a month later, the ulcer measured 1.5 × 1.5 cm with an indurated area. The LL was epithelialised and tumour free at his third follow-up, a month later. A final evaluation, conducted 2 years after treatment, revealed that the patient was asymptomatic and disease-free. However, mild atrophy and skin hypopigmentation were observed as long-term side effects of the treatment. The patient stated that he was pleased with the results and aesthetic outcome of his procedure (Figure 5).

## Discussion

In the present case, LC was detected at a relatively early stage, so surgery or radiotherapy are both viable options with similar survival rates in the case of tumours larger than 2 cm. However, it has been shown that surgery for LC can cause undesirable side effects such as scarring, loss of lip function and microstomia [11–12]. The recommendations of the Groupe Européen de Curiethérapie (GEC) and the European Society for Radiotherapy & Oncology (ESTRO) for the use of HDRIB are LC with a thickness ≥ 5 mm with an irregular surface, 8–12 mm distance between catheters that must be at least 3 mm below the skin surface to avoid complications. Additionally, it is necessary to use CT to define the CTV [8].

Radiotherapy for LC may be administered using an external radiation source, by low-dose-rate interstitial brachytherapy (LDRIB), pulsed dose rate (PDR) brachytherapy [13] or by HDRIB (Table 2) [12]. The difference between these radiotherapy treatments is activity of the implants (0.4–2 Gy/h, 2–12 Gy/h and > 12 Gy/h, respectively) and the length of time the patient is exposed to the radioactive sources [12]. Indications for their use are similar and include T1 to T2-staged tumours, positive surgical margins and post-surgical recurrences. They are contraindicated in cases with bone involvement or significant tissue loss [4]. Additionally, BT does not seem to be indicated for adenocarcinomas, lymphomas, melanomas and sarcomas, except for rhabdomyosarcomas [12]. In cases of LC with advanced tumours or with lymph node involvement, external radiotherapy that includes the primary tumour and neck with a dose up to 45 to 50 Gy followed by BT is the preferred route [4].

HDRIB has the same efficacy as LDRIB for LC. In a retrospective study, where 103 patients were followed for an average of 3.1 years, Ghadjar *et al* [14] found that there was no difference in survival rate or acute or long-term toxicity between either technique. Guinot *et al* [15] compared the same methods in a similar study that involved 99 patients. Likewise, the study resulted in similar conclusions: long-term local control and disease-free survival were similar. However, fewer complications were observed with HDRIB treatment. An additional advantage is that HDRIB allows for optimising dose distribution [15].

In HDRIB, the equivalent dose per fraction is usually 4.5–5 Gy in approximately nine fractions distributed at two fractions daily for 5 days. The planning of HDRIB with ^192^Ir follows the Paris System. HDRIB is a useful treatment option for patients with inoperable T1–T2-staged tumours or those who reject surgery. Additionally, HDRIB offers excellent aesthetic and functional results, low toxicity and local control between 88% and 100% of cases [15–18]. Acute toxicity is most commonly grade 1 and requires topical medicine. Long-term side-effects include hypopigmentation, telangiectasias and in some cases a certain degree of fibrosis. However, these are very rare: over 90% of patients treated with HDRIB for LC have satisfactory functional and cosmetic results, and most patients who underwent the procedure would recommend it [8, 19, 20].

## Conclusions

HDRIB has been proven to be useful in treating LC and other tumours of the oral cavity either alone or after surgery with compromised margins, or with external radiotherapy.

The present study adds to the growing body of evidence that HDRIB using ^192^Ir is effective in patients with early-stage LC who do not wish to undergo surgery or have comorbidities that make surgical revision of the tumour unacceptable. Curative EQD2 dose ranges between 50 and 60 Gy. In the present case, an EQD2**_ (α/β_**_ = 10)_ dose of 56 Gy in HDRIB resulted in disappearance of the cancerous lesion with few side effects, minimal toxicity and acceptable aesthetics.

Furthermore, this case reports one of the first treatments of LC with HDRIB in Peru, thus overcoming challenges of administrative inertia and logistics. This case also shows that good doctor–patient collaboration can occur in under-resourced environment and patients can receive a high standard of care. This requires considering the patient’s desires and likely outcomes of treatment. Although surgical resection is still practiced in similar cases, the successful treatment of this patient has already encouraged doctors to more regularly use HDRIB as an alternative to surgery based on patient preference or when comorbidities complicate surgery.

## Conflicts of interest

The authors declare that they have no economic or non-economic conflicts of interest in the publication of this article.

## Funding statement

The authors received no funding for the research and publication of this study.

## Authors’ contributions

PTQ – radiation oncologist who performed the BT procedure and supervised the treatment of the patient.

YZ – radiation oncologist who contributed to writing the discussion section.

LJFR – radiation oncologist who recompiled the case, wrote the manuscript and prepared the figures.

ARC – radiation oncologist, BT specialist who mentored all participants, senior researcher.

## Figures and Tables

**Table 1.** Treatment plan parameters.

Treatment details	HDRIB to the lower left lip
BT dosage	45.0 Gy to the tumour in nine fractions, 5.00 Gy per fraction. Dose planned to cover the PTV is 90% of 45 Gy = 40.5 Gy total dose in 4.5 Gy per session.EQD2 for 100% = 56 Gy, for 90% is 45 Gy, assuming α/β = 10
Target definition	Gross tumour volume = CTV CTV + 1 cm = PTV PTV volume = 9.18 cm^3^
Number of catheters	5
Treatment plan parameters	D_90_ = 4.50 Gy V_100_ = 1.19 cm^3^ V_150_ = 1.10 cm^3^ V_200_ = 0.64 cm^3^
Calculated dosage to organs at risk per session	Lower jaw: 0.7895 Gy Spinal cord: 0 Gy Left eye: 0.2588 Gy Right eye: 0.1200 Gy Thyroid gland: 0 Gy Gingiva: 0.9986 Gy

PTV: planning target volume

**Table 2.** Comparison of different BT alternatives in LC treatment.

Characteristics	BT type		
	LDRIB	PDR	HDRIB
Dose activity (Gy/h)	0.4–2	2–12	>12
Indications	Tumours T1–T2. Positive surgical margins Post-surgical relapse.	Tumours T1–T3. Positive surgical margins Post-surgical relapse.	Tumours T1–T2, T4 (low-volume) Positive surgical margins Post-surgical relapse.
Contraindications	Bone infiltration	Bone infiltration	Bone infiltration
Local control (%) in 5 years	94%–96.6%	95%	>95%
